# Generative Self-Attention for Adaptive Real-Time Traffic Event Detection in Next-Generation Intelligent Transportation Systems

**DOI:** 10.3390/s26195999

**Published:** 2026-09-22

**Authors:** Sakshi Verma, Sushil Kumar Tiwari, Vishal Krishna Singh, Rajkumar Singh Rathore, Weiwei Jiang

**Affiliations:** 1 Department of IT, Indian Institute of Information Technology, Lucknow 226002, India; rcs201001@iiitl.ac.in (S.V.); stiwari@iiitl.ac.in (S.K.T.); 2 School of Computer Science and Electronic Engineering, Colchester Campus, University of Essex, Colchester CO4 3SQ, UK; 3 Department of Computer Science, School of Technologies, Cardiff Metropolitan University, Cardiff CF5 2YB, UK; 4 School of Information and Communication Engineering, Beijing University of Posts and Telecommunications, Beijing 100876, China

**Keywords:** data stream, meta-path, higher information network, incremental learning, graph neural network

## Abstract

Event detection on real-time traffic data is crucial for transportation systems, enhancing safety in autonomous systems and the resilience of urban mobility infrastructure. However, the complex, heterogeneous traffic stream data and spatial-temporal patterns pose a significant challenge to existing methods, which often fail to capture higher-order interactions among nodes and edges and semantic context. This paper proposes a generative, self-attention model layered with two stacked content-based layers based on a framework that incrementally handles the process of traffic stream networks. Experimental analysis on benchmark event -detection dataset demonstrates the effectiveness of the proposed methodology, attaining peak performance values of 0.80 in Normalized mutual information(NMI), 0.73 IN Adjusted Mutual Information (AMI), and 0.72 in Adjusted Rand Index(ARI) across the incremental block of heterogeneous data, the proposed method consistently outperforms state-of-the-art baselines.

## 1. Introduction

Monitoring traffic events in real-time is essential for handling situations that impair road safety, cause congestion, and interfere with traffic flow. Especially in crowded metropolitan areas, efficient deployment is critical to traffic control, emergency response, and smart city infrastructure [1,2,3]. Accidents and bad weather are disruptions that frequently worsen delays, pollution, and financial losses. Transportation networks are more resilient and efficient when detection occurs quickly and domain-specific adaptation strategies, such as model compression techniques, specialized dataset construction, and cloud-edge-vehicle collaborative deployment for LLMs [4]. However, because traffic networks are dynamic and complicated, there are still significant problems. Traditional detection techniques frequently ignore the complex spatiotemporal connections required for precise event identification in favor of surface-level data. Scalability, dynamic learning, and a thorough comprehension of real-time traffic patterns are all requirements for advanced models.

The intrinsic heterogeneity and dynamic nature of traffic networks consisting of various entities (such as cars, road segments, and traffic lights) and dynamically changing interactions present a difficulty for real-time traffic event identification. These networks are constantly evolving due to road conditions, vehicle density, and speed changes. Conventional detection techniques mainly depend on surface-level characteristics, including vehicle counts, average speeds, and road usage frequencies. These techniques include rule-based models, statistical analysis, and incremental learning approaches. Although these methods offer some helpful information, they frequently need to focus more on the intricate spatiotemporal relationships and contextual elements that significantly impact traffic events, which can result in delayed or incorrect event identification.

Recent advancements in real-time traffic event detection have leveraged various methods, including the analysis of social media streams and the application of deep learning models. The author [5] used a DistilBERT-GNN, an incremental event detection method proposed that combines DistilBERT and graph neural networks (GNNs) to provide real-time contextual understanding with GNNs’ ability to capture evolving relationships in social media networks, to increase the detection. and The rapid growth of social media platforms to identify recent developments and comprehend public opinion in several languages has increased due to the quick expansion of social media platforms. A multilingual polarization detection [6] method spanning 22 languages was proposed by the author. The authors use GPT-4o-mini and three synthetic data techniques (direct generation, paraphrasing, and contrastive pair construction) with a multi-stage quality filtering pipeline that incorporates embedding-based deduplication. Additionally, there are issues with explainability, cross-lingual generalization, synthetic data quality, scalability, and idea drift with current approaches. Graph Neural Networks (GNNs) [7] are particularly well-suited for modeling dissimilar traffic data. GNNs excel at modeling the interactions of diverse nodes and edges in traffic networks and can capture the temporal (e.g., the progression of vehicles over time) and spatial (e.g., traffic segments and traffic control devices) aspects of traffic networks. Traffic event detection can be further improved by using Heterogeneous Information Networks (HINs), which comprise multiple objects and multiple relations and therefore provide a more detailed representation of traffic patterns. Drawing on the GNN-based techniques and other advanced methods, various drawbacks still exist that pose critical problems for real-time traffic event detection. These drawbacks include the challenge of efficiently implementing existing wet models. For example, traffic incident detection with urban Twitter data has adopted a RoBERTa-based model, an improved version of BERT with particular task performance optimizations. The model shows great accuracy at classification with the drawback of spatial accuracy, among other social media data classification obstacles [8]. Because of this, the model is limited for many use cases.

Because of the complex relationships of data with time and space, the MegaGraph Convolution RNN has adopted Meta-Graph Learning for transportation prediction [9]. This model has shown significant success with the METR-LA and PEMS-BAY data sets. The model is still limited by the cost of computation and flexibility in real time and has the same issues of broader use outside of these data sets.

A RoBERTa-based model, which is a more advanced version of BERT that has been fine-tuned for specific tasks, has also been used on Twitter data from different cities to identify traffic issues like accidents and congestion. This model has been shown to have great accuracy for classification. However, the model’s broader applicability is limited by the noise stemming from Twitter data and a lack of geographic accuracy.

The spatio-temporal Meta-Graph Learning technique resolves some of these limitations by focusing on the complicated spatio-temporal correlations in traffic forecasting. This technique is part of the Meta-Graph Convolutional Recurrent Network (MegaCRN). MegaCRN has shown superior performance to models on the METR-LA and PEMS-BAY datasets. As with the other techniques, the costs associated with computation and delays in real-time performance still make MegaCRN hard to put into practice. Investigating real-time traffic detection systems, including those that monitor the Italian road network, support analysis of Twitter data using Support Vector Machines (SVMs) [10]. Even with such innovative frameworks, noise, and ambiguity in the social media content affect the accuracy of detection. Another improvement in this field, the Real-Time Monitoring Architecture (RTMA) allows extraction of traffic events from social media, utilizing sequence labeling [11] and combining knowledge extracted from the domain. While this method has shown improvement, biases in user-reported data and the challenge of processing large amounts of unstructured, continuous real-time data remain.

Most of the methods used for event detection in heterogeneous information networks (HINs) are mostly concentrated on static networks [12]. On the contrary, real-life HINs are dynamic and consist of the constant addition or removal of nodes and edges. This is where the DyHNE model [13] comes in.

Other HGT methods focus on heterogeneous graph representation and large-scale learning, but do not address the incremental, real-time traffic data stream or interdependencies of nodes in higher order.Early methods, such as TF-IDF with SVM or Naïve Bayes, for Twitter traffic event detection suffer from sparsity and loss of word order [14]. Social media Twitter data analytics using NLP, topic modeling, and sentiment analysis on large-scale extraction is difficult to capture higher-order neighborhoods and remains limited by noisy and biased data [15]. Recently added Random Forest and basic GCN methods [16] struggle with the irregular topology of urban road segments and severe data imbalance in crash prediction. The Semantic-Reconstructed-based Graph Transformer Network [17] for event detection is designed for static semantic textual event detection and does not address spatiotemporal dynamics, evolving heterogeneous traffic graphs, or higher-order neighborhood nodes, which are highly involved in stream-data detection.

The DyHNE model uses proximate meta-paths to capture dynamic and intricate semantics. While this model has been useful, many of its own unresolvable issues still persist. Other models, such as the Heterogeneous Hyperedge Convolutional Network (HHCN) [18], employ meta-structures along with hyperedges to frame relationships but struggle to retain information. On the other hand, the Heterogeneous Attentive Entity Graph Neural Network [19] captures complex semantics using the self-attention model, but has large latency and computing costs associated with it. Among incremental learning models, the Knowledge-Preserving Graph Neural Network (KPGNN) is a notable solution for detecting social events by continuously analyzing incoming social streams. While effective, KPGNN struggles with higher-order node embeddings and deeper semantic representation. A more recent innovation, the “Lime: Low-Cost and Incremental Learning for Dynamic Heterogeneous Information Networks” framework [20], combines incremental learning with heterogeneous attribute enhancement to improve real-time event detection. Despite its promise, challenges involving scalability, sensitivity to noisy data, and computational overhead still need to be addressed.

The proposed work aims to enhance accuracy in real-time traffic event detection by leveraging a self-attention mechanism to thoroughly examine content-based node interactions within the HAE framework, coupled with incremental learning. This approach enables an in-depth analysis of relationships among both direct and higher-order neighbors, resulting in improved detection accuracy. Additionally, this method enhances the system’s affordability, efficiency, portability, and maintainability.

The contributions of the proposed work are summarised as follows:

- The proposed method preserves relationships between nodes and their neighbors of higher order in dynamic heterogeneous networks by employing a heterogeneous attribute enhancement (HAE) technique. This method combines semantic and content-based layers and improves real-time event detection in intricate information environments by guaranteeing accurate extraction of pertinent node properties.
- Due to the semantic diversity and richness of the data, implementing a graph neural network (GNN) inside the HAE technique for heterogeneous graphs presents challenges.
- A real-time event detection algorithm tackles challenges stemming from node heterogeneity, where each node type exhibits unique features.
- A comprehensive comparative analysis highlighting the accuracy improvements achieved by the proposed method.

The rest of the paper is organized as follows. Section 2 describes the problem related to real-time event detection. Section 3 reviews the system model relevant to the proposed model. Section 4 outlines the methodology of the proposed model. Section 5 discusses the experimental results, and the paper concludes in Section 6.

## 2. Problem Description

Consider a real-time traffic event detection system using a heterogeneous information network (HIN) can be represented by a sequence of parameters ($D_{1}$, $D_{2}$, …, $D_{t}$), where at time *t*, The network D = (v,e,w). is composed of multiple types of entities: vehicles, road segments, traffic lights, and junctions. These entities reflect the interconnected structure of traffic systems, where various components interact to shape the overall traffic dynamics. As illustrated in Figure 1, vehicles V1 and V2, V3, V4, etc, are interconnected via links and junctions, and notably, Blue-colored nodes are likely used to represent dynamic elements or interactions in the traffic system, and Dashed edges are likely used to represent higher-order or indirect links, such as Alternative routes or potential collisions with other vehicles. The Figure 1 shows that two vehicles V1, V3, may navigate through the same junction, $J_{1}$, while a third vehicle, V3, traverses a different junction, $J_{2}$. Based on the meta-path illustrated in Figure 2, V2 and V3 are classified as neighbors of V1. Although no direct interaction occurs between V2 and V3, an indirect connection exists through a shared association with V1. Analyzing content-based interdependencies necessitates a comprehensive examination of higher-order neighbor connections extending beyond direct links between nodes, since conventional traffic analysis methods often overlook the intricate complexity of such relationships. The dynamic HIN framework for traffic event detection can be formalized as follows: Let $A=V_{1},V_{2},V_{3}$ represents set of vehicles, $C=J_{1},J_{2}$ represents junctions and $P=R_{1},R_{2}$ represents the road segments. The Meta-Path P1 can be represented as:

(1) $$V2\overset{R_{1}}{\to }V1\overset{R_{2}}{\to }V3$$

- Vehicles V1 and V2 travel on road R1 and pass through junction $J_{1}$.
- Vehicles V1 and V3 travel on road R2 and pass through junction $J_{2}$.

However, a comprehensive analysis reveals that the accuracy of these methodologies degrades significantly due to the following key challenges:

- Diversity of Node Types: Traffic networks are composed of heterogeneous node types, such as vehicles, junctions, road segments, and traffic signals, each with distinct feature sets. This heterogeneity poses significant challenges when constructing a graph neural network (GNN) that can effectively manage and integrate the diverse data attributes.
- Complex Relationships in Heterogeneous Traffic Graphs: Nodes within Multiple linkages, including meta-paths and meta-graphs, connect diverse traffic graphs. encapsulating the diverse interactions inherent in traffic networks.
- Preservation of Node-Specific Information: Capturing the unique information associated with individual nodes while concurrently modeling higher-order relationships within a dynamic and evolving traffic network presents substantial difficulties.

The inherent heterogeneity of entities, such as vehicles, roads, and junctions, implies that each entity type possesses distinct features. Developing a GNN for heterogeneous traffic networks is particularly challenging due to the complexity of interactions and the necessity to extract meaningful relationships across diverse node types. Vehicles may be connected through various meta-paths and meta-graphs, and each vehicle could have multiple neighbors sharing the same meta-path. Consequently, it becomes crucial to distinguish between these neighbors and prioritize the most relevant connections. Additionally, preserving the individual information of vehicles while simultaneously capturing their higher-order dependencies in a dynamic traffic environment introduces further complexity.

## 3. Preliminary

This paper defines essential concepts such as data streams, heterogeneous information networks (HINs), network schemas, meta-paths, meta-graphs, and the incremental learning process for real-time event detection.

Concepts 3.1. A data stream is an infinite, dynamic, continuous, and time-sensitive. Each data block $k_{i}$ encompasses all data between time points $t_{i}$ and $t_{i+1}$. Real-time processing of data streams is necessary to reflect incoming data in newly created outputs in a matter of seconds with minimal delay.

Concepts 3.2. A HIN is a graph G = (*v*, *e*) consisting of multiple types of entities and relationships connected by different types of relations. In addition to the graph structure of the data association, HIN captures higher-order semantics of the data, allowing for a richer understanding of interactions. As illustrated in Figure 1, different entities (e.g., vehicles, roads, and junctions) engage in various traffic patterns, each influenced by distinct environmental factors and conditions.

Concepts 3.3. Meta-Path m is based on a network schema m, which is denoted as

$$m=A_{1}\overset{R_{1}}{\to }A_{2}\overset{R_{2}}{\to }\cdots \overset{R_{i}}{\to }A_{i+1},$$

where $A_{1},A_{2},\ldots ,A_{i+1}\in A$ represent the node types and $R_{1},R_{2},\ldots ,R_{i}$ are the relations between them. The composite relation is given by.

And the composite relation

$$R_{1}\circ R_{2}\circ \cdots \circ R_{i+1}$$

between node types. Make sure that various meta-paths explain semantic links from different perspectives, as shown in Figure 3b.

Concepts 3.4. A meta-graph constitutes a directed acyclic graph (DAG) comprising several shared blocks and meta-paths as shown in Figure 3c. It is defined by a network schema m = (*v*, *e*), where *v* represents the nodes and *e* represents the edges in the graph. Meta-graphs can be portrayed as DAGs containing a single source node and one corresponding “sink” node.

Concepts 3.5. Incremental learning is shown in Figure 4 that continuously updates a model using new and old data, balancing knowledge assimilation and retention. Ensures adaptability in real-time event detection by mitigating forgetting while processing evolving data streams.

Table 1 summarizes the symbols and their descriptions used throughout this study.

## 4. Methodology

The following section presents a comprehensive description of the proposed method. The key characteristics of the proposed model are given in Section 4.1. Section 4.2 discusses a new Higher-Order Attribute-Enhancing (HAE) model intended to capture complicated semantics and exploit the effect of higher-order neighboring nodes. Section 4.3 provides a comprehensive overview of the algorithmic method.

### 4.1. Proposed Architecture

The proposed approach includes all three stages of an incremental learning algorithm, such as pretraining, detection, and maintenance. As shown in Figure 5, the process consists of the following stages:

- Pretraining: Data collected from online streams, particularly from Twitter, are preprocessed. The preprocessing stage consists of transforming raw data into a dynamically changing heterogeneous information network (HIN), which is done using meta-paths, and then calculating the event similarity measurement to pass into the next stage of detection.
- Detection: After pre-processing, data goes to the next phase – detection. At this stage, the HAE method is used for embedding of the nodes. This method uses two layers: semantic-based and content-based. While the semantic-based layer reveals complex relations between nodes, the content-based layer detects complex semantics. The HAE algorithm was determined by its capacity to represent higher-order neighbors with the same attributes. The next stage of the process is the refinement of the obtained node embeddings.
- Maintenance: This stage involves removing irrelevant nodes and continuing model training. After completing the three preceding steps, the data advances to the event detection stage, where the final event detection outcomes are generated.

### 4.2. Heterogeneous Attribute Enhancing (HAE)

The HAE model consists of an initial semantic-level approach and a series of content-based self-attention mechanisms.

Each component’s output serves as input to the subsequent one, as depicted in Figure 6. The semantic-based approach enhances the features of input nodes by analyzing semantic and content-based interactions among nodes using the Graph Convolutional Network (GCN) technique. Subsequently, the content-based self-attention mechanism refines the properties of the input nodes using the graph attention technique (GAT).

#### 4.2.1. Semantic-Based Convolution Layer Approach

Enhances the semantic richness of input nodes by incorporating them into different meta-paths and meta-graphs, as illustrated in Figure 6. This semantic-based method evaluates the multiple semantic forms of each pair of target nodes by measuring the similarity between their respective node components. The process includes the following steps:

- Single Meta-Path Similarity Calculation: A single meta-path is first used to calculate the node similarity between two nodes.
- Multi-Semantic Structure-Based Similarity Examination: Subsequently, the multi-semantic structure-based node similarity is assessed. This is achieved by manipulating commuting matrices and parameterizing the semantic structure weights based on the similarity. For a given meta-path M = $X_{1}$, $X_{2}$, $X_{3}$, $X_{4}\ldots \ldots \ldots X_{K}$ and calculating the commuting matrix is defined as
  $$C_{M}=W_{X_{1}X_{2}}\cdot W_{X_{2}X_{3}}\cdot \ldots \cdot W_{X_{K-1}X_{K}}$$
  where $W_{XiXi+1}$ is the adjacency matrix representing the relationship between $X_{i}$ and $x_{i+1}$ node types. This approach creates an adjacency matrix, denoted by A in Figure 6.
- Combining Semantic Data: The proposed method integrates diverse semantic data from meta-paths and meta-graphs using sub-layers, guided by propagation rules. By incorporating these steps, the framework effectively utilizes the rich semantic properties of the input nodes, thereby enhancing the overall semantic-based node similarity assessment. The proposed architecture with sub-layers following the propagation rules followed by GCN:
  (2) $$H^{i+1}=\alpha \left(D^{-1/2}AD^{-1/2}H^{I}W^{\left(I\right)}\right)$$

#### 4.2.2. Prerequisite

Definition: Node Similarity (NS) Based on Instances of Semantic Structure- Given a collection of meta-path and meta graph $X={x_{n}}^{N}$ that start and end with target type $K_{t}$ the node similarity between $V_{ti}\in K_{t}$ and $V_{tj}\in K_{t}$ is defined as

(3) $$NS\left(v_{ti},v_{tj}\right)=\sum_{n=1}^{N}\frac{2C_{X_{n}}\left(i,j\right)}{C_{X_{n}}\left(i,j\right)+C_{X_{n}}\left(j,j\right)}$$

#### 4.2.3. A Content-Based Self-Attention Layer

Incorporating a content-based self-attention mechanism involves leveraging node interactions based on content to amplify the characteristics of the input node using the Graph Attention Network (GAT).This concept is exemplified in Figure 6. The steps include:

- Generation of Adjacency Matrix: This mechanism begins with the creation of an adjacency matrix derived from a multi-semantic structure, labeled as A in Figure 6.
- Attention to Node Neighbors: The mechanism attends to the content of neighboring nodes, allowing for a refined aggregation of information. The term t pays attention to each node’s neighbors to compute that node’s representation. By adopting this approach, the event detection model can effectively capture intricate semantics present in the input data, leading to a notable enhancement in its performance.
  The first step is to create an adjacency matrix called $M^{\prime }M^{\prime }\in R^{N*N}$, N represents the number of meta-paths and meta-graphs that start and stop at the target node type $A_{t}$.
  $M^{\prime }$ is a binary matrix, and $M_{IJ}^{\prime }=1$ only if there is at least one occurrence of a meta-path or meta-graph connecting nodes $v_{ti}$ and $v_{tj}$.
  For a given node $v_{ti}\in A_{t}$, the neighborhood $N(v_{ti})$ is defined as:
  $$N\left(v_{ti}\right)=\left\{v_{tj}|M_{ij}^{\prime }=1\right\}$$
  where $M^{\prime }$ is the adjacency matrix representing the relationships between nodes, and $M_{ij}^{\prime }=1$ indicates a direct connection between nodes $v_{ti}$ and $v_{tj}$.
- Capturing Intricate Semantics: This approach effectively captures complex semantics present in the input data.
- Combining Semantic Data: The proposed method integrates diverse semantic data from meta-paths and meta-graphs using sub-layers, guided by propagation rules. The semantic structure based on the neighbourhood, represented by M′, was the focus of a veiled attention. This mechanism is described as follows: a: R d’*d’—R, where d’ stands for the content-based approach’s output dimension. shows one non-linear feed-forward layer with a single attention coefficient output.
  (4) $$g_{ij}=\alpha \left(Wv_{ti},Wv_{tj}\right)=\sigma (\alpha^{T}[\left(Wv_{ti}\right)||Wv_{tj}])$$

#### 4.2.4. Attention Normalization

Determine the normalized attention coefficient using the SoftMax function:

(5) $$a_{ij}=softmax\left(g_{ij}\right)=\frac{exp(\sigma (\alpha^{T}[WV_{ti}||Wv_{tj}]))}{\sum_{n\in N_{i}}exp(\sigma (\alpha^{T}[(Wv_{ti}||Wv_{tj}]))}$$

- Performance Enhancement Incorporating content-based self-attention significantly improves the model’s performance in event detection by accurately understanding and utilizing semantic nuances. Then apply attention to its neighbors using the normalized attention coefficients and calculate the output of $v_{ti}$.
  (6) $$V_{ij}^{\prime }=\sigma \left(\sum_{v_{tj}\in N_{t}}\alpha_{ij}Wv_{ti}\right)$$

#### 4.2.5. Multi-Head Attention

Further, apply multi-head attention to sustain the learning process then again calculate the output $v_{ti}$ as the final representation.

(7) $$V_{ij}^{\prime }={∥}_{h=1}^{H}\sigma \left(\sum_{v_{tj}\in N_{t}}\alpha_{ij}W^{h}v_{ti}\right)$$

### 4.3. Two Stacked CAL Architecture

As shown in Figure 7, A CAL elevates each road junction (V1, V2,V3, V4, V5, V6) embedding with the embedding of its first-order semantic-structure-based neighbors. In the first stack layer, CAL elevates V1 with V2 and V3, and V2 with V1 and V4. The second stack then repeats the process and elevates V1 and V2. At that point, V2 is enhanced with V4 by the first stack CAL and, therefore, the second stacked CAL indirectly elevates V1 with V4. The enhancing process is guided by a process-specific loss function. The loss function can be formalized as:

(8) $$L_{T}L=-\sum y_{l}\log\left(\mathrm{softmax}\left(w_{t}+b\right)\right)$$

### 4.4. Incremental Update Mechanism

Suppose $H_{t}$ denote the learned representation at traffic block *t*. When a new block $G_{t+1}$ arrives, the previous representation is retained and updated using the newly observed information:

$$H_{t+1}=\left(1-\lambda_{t}\right)H_{t}^{*}+\lambda_{t}{\hat{H}}_{t+1},$$

where $H_{t}^{*}$ is the retained historical representation, ${\hat{H}}_{t+1}$ is the representation learned from the new traffic block, and $\lambda_{t}\in \left[0,1\right]$ controls the contribution of new information.

### 4.5. Proposed Algorithm

The proposed model functions incrementally, as depicted in Figure 5 and Figure 6, with its operation detailed in Algorithm 1. Initially, a data stream sequence, denoted as $d_{1},\ldots ,d_{n}$, is established within a window size *w* and divided into layers $l_{1},l_{2},\ldots$. The process begins by constructing an input sequence graph if data are available; otherwise, the data block graph is updated. If the current time is non-zero, event detection is performed on $D_{t}$ using Equation (6). Following this, message clustering is applied to the data.

**Algorithm 1** Training Process for Heterogeneous Graph Model
  1:**Input:** A fixed data stream (S = $D_{0},D_{1},D_{2},$ …), where $\left(D_{t}\right)$ denotes the data received at time step (t); a sliding window of size (w); (L) graph layers; and (B) mini-batches used for incremental processing.  2:**Output:** A sequence of detected event sets (E = $E_{0},E_{1},E_{2},\;ldots$), where $\left(E_{t}\right)$ represents the set of events identified at time step (t).  3:**for** t=0,1,2,3,… **do**  4:      **if** t==0 **then**  5:            F← Combine node embeddings with respective attribute embeddings to form the initial heterogeneous graph (Section 4.2)  6:      **else**  7:            F← Update $D_{t}$ into block graph (Section 4.2)  8:            **if** t!=0 **then**  9:                  Detect event from $D_{t}$10:            **end if**11:            **for** l=0,1,2,3,… **do**12:           $$V_{ij}^{\prime }={∥}_{h=1}^{H}\sigma \left(\sum_{v_{tj}\in N_{t}}\alpha_{ij}W^{h}v_{ti}\right)$$13:                  $V_{T}\leftarrow V_{T}^{M}$14:            **end for**15:            $E_{t}\leftarrow$ Message Clustering16:            **if** t/w==0 **then**17:                  Initiate pre-training phase for the model18:                  **if** t!=0 **then**19:                        F← Remove message block (see Figure 7 and Section 4)20:                  **end if**21:                  **for** b=0,1,2,3,… **do**22:                        Train in blocks23:                        $G_{b}\leftarrow$ Sampling of a stream data set24:                        **for** l=0,1,2,3,… **do**25:                   $$V_{ij}^{\prime }={∥}_{h=1}^{H}\sigma \left(\sum_{v_{tj}\in N_{t}}\alpha_{ij}W^{h}v_{ti}\right)$$26:                              $V_{T}\leftarrow V_{T}^{M}$27:                        **end for**28:                  **end for**29:            **end if**30:            i←i+231:            L← Loss function32:            $L_{T}L=-\sum y_{l}\log\left(\mathrm{softmax}\left(w_{t}+b\right)\right)$33:            $V_{T}\leftarrow$ Calculate summary and corrupted34:            Perform backpropagation to update parameters35:      **end if**36:
**end for**



If the model determines that the window size exceeds capacity while time remains nonzero, obsolete messages are removed from the data stream window. Subsequently, all layers of the data stream are applied, and a loss function is employed to predict the desired output. The model is then updated accordingly.

The life cycle of the model consists of three phases (pretraining, monitoring, and maintenance) as described in the algorithm and is shown in Figure 8. During the pretraining phase, an initial Block Graph is made, and an initial model is trained. As new data flows in and events are recognized, the model updates the Block Graph in the monitoring phase. The model is continuously active on the Blocks until the maintenance phase. In the maintenance phase, retraining of the model is done on the newest data, while obsolete Blocks of the Graph are removed. This allows the model to maintain its relevance and knowledge and optimizes its performance.

## 5. Experiments and Results

This section describes the framework of the experiments regarding the selection of the datasets, comparison of the baselines, the criteria of the evaluations, and the settings of the parameters. The data analyzed during this study is explained in Section 5.1. In Section 5.2, the methods used to establish the comparison baselines are described. Evaluation metrics are explained in Section 5.3. Section 5.4 describes the incremental analysis. A performance analysis is described in Section 5.5.

### 5.1. Dataset

Much effort was made to validate the model on multiple large public datasets. The focus was mostly on the Twitter dataset [13]. This dataset contains multiple message blocks on traffic with incremental timestamps, containing 68,841 tweets on 503 different events with traffic. The dataset was pruned so that duplicates, tweets that could not be accessed, and tweets that contained multiple events were removed. This resulted in a dataset of 10,242 messages on 154 events. The dataset created is substantially larger than datasets used in other similar works.

### 5.2. Baselines

To evaluate the effectiveness of the proposed methodology for real-time traffic event detection, a comparative analysis was performed against various baseline methods, including BS-GAT [10], CNN and LSTM [14], SRGTNED [17], KPGNN [21], Word2Vec [22], LDA [23], WMD [24], PP-GCN [25] and SHetGNN [26] All methods demonstrate different methodologies for event detection. This analysis focuses on message representation learning and similarity measurement.

### 5.3. Evaluation Metrics

The performance evaluation of all models involved the application of several metrics, including normalized mutual information (NMI), adjusted mutual information (AMI), and adjusted Rand index (ARI). NMI was employed to measure the information extracted from the distribution of predictions relative to the distribution of actual ground-truth labels, while AMI measures the mutual information between two clustering adjusted for the chance. ARI considers all prediction-label pairs and adjusts for chance. These metrics are commonly used in evaluations of social event detection methods.

### 5.4. Incremental Analysis

To detect traffic events in real-time, this section evaluates the suitability and significance of Twitter data using an incremental detection technique. Assessing the accuracy of Twitter data in reflecting real-world traffic conditions is crucial, considering potential constraints such as driver/user activity and the average number of users per vehicle.

To simulate a social stream, the Twitter dataset was segmented by date. Tweets from the first week were used to generate the initial message block, M0. Subsequent message blocks, M1, M2, and so forth, up to M16, were constructed using tweets from the following days. However, since not all drivers submit traffic-related tweets—potentially introducing bias—the accuracy of this data depends on user reporting behavior. Table 2 presents the statistics of the resulting social stream, aiding in the evaluation of the precision and representativeness of Twitter-based traffic analysis.

The Performance of the method in terms of NMI, AMI, and ARI is illustrated in Figure 9, Figure 10, and Figure 11, respectively, through various strategies for update-maintenance operation. The large value of NMI and AMI provide consistency in the output of the algorithm, leading to a decreased number of erroneous event detections and enhanced decision-making in a time-critical situation. Our approach takes into account the semantic aspect as well as the interdependency of higher-neighborhood nodes based on the content. The variation seen in the graph of ARI (see Figure 11) shows the adaptability of our approach to changes in traffic environment dynamics.

Unlike conventional approaches, our method efficiently integrates the newest messages while maintaining compatibility with previous updates. This enhances stability and adaptability, as reflected in the smoother performance trends.

### 5.5. Performance Evaluation

#### 5.5.1. Comparison with Baseline Methods (NMI, AMI, ARI)

The incremental results of the detection of social events, detailed in Table 2, Table 3 and Table 4, demonstrate that the proposed method outperforms the baseline methods in all message blocks in terms of Normalized Mutual Information (NMI), Adjusted Mutual Information (AMI) and Adjusted Rand Index (ARI). The proposed approach achieves significant relative performance gains over Word2Vec, WMD, and LDA, with improvements of 2–40% in NMI, 13–52% in AMI, and 7–44% in ARI over Word2Vec; 167% in NMI, 663% in AMI, and 4–66% in ARI over WMD; and 3–70% in NMI, 6–59% in AMI, and 3–69% in ARI over LDA and Proposed Method significantly outperforms BS-GAT in all metrics, especially in AMI (+77%) and ARI (+114%), demonstrating stronger cluster consistency and robustness in incremental settings.

Compared to KPGNN, the Proposed Method shows consistent but moderate gains, indicating that both models are high-performing, but your approach maintains a stable improvement (3–6%) across NMI, AMI, and ARI. These improvements are largely attributed to the incorporation of structural information, often overlooked by other methods, enhancing the model’s ability to capture complex relationships in social data streams.

#### 5.5.2. Ablation Study: Inference Time, Memory, Scalability and Network Size

During the maintenance stage, the method was trained using one minibatch, and its training time and memory usage were recorded. Figure 12 shows the ablation study: Inference time, Memory, scalability, and network size. These results indicate the computational requirements of the proposed framework and its suitability for processing streaming data in real-time event detection applications.

#### 5.5.3. Comparison with Other Methods (Accuracy, Precision, Recall)

Comparative analysis of the superior performance of the proposed approach compared to SHetGNN [26], SRGTNED, and CNN and LSTM. The SHetGNN algorithm achieves 0.9791 accuracy, 0.9734 precision, and 0.9725 recall, SRGTNED achieves 0.793 preision, 0.799 recall and 0.972 F1 score and CNN and LSTM achieves 0.976 precision, 0.962 recall and 0.972 F1 score while the proposed approach achieves 0.9855 accuracy, 0.9843 precision, and 0.9887 recall. This higher performance is attributable to improved feature representation, self-attention capabilities, strict data manipulation, and incremental learning that improve instance classification and the identification of useful information within the data, as shown in Table 5.

#### 5.5.4. Methodological Comparison

The proposed method for detecting events takes advantage of similarity based on the basis of a content-based layer, semantic convolution, and attention-based self-learning in order to perform better and thus make use of higher-order neighborhood node information as well as user-guided structuring of data. In contrast to SHetGCN [26], which uses short walks according to meta-paths in order to classify domain nodes inductively in Heterogeneous Information Networks (HINs), the suggested model works in real time and detects events from streams of data.

#### 5.5.5. Conclusion on Performance Superiority

This paper proves to be better than other baselines, as it is able to efficiently capture spatiotemporal information from the stream data due to the structural information provided by the social stream. Such an approach allows for better performance in real-time environments.

## 6. Conclusions

The proposed approach effectively detects events in streaming data by utilizing a self-attention mechanism to assess content-based interactions among nodes thoroughly. This method enables a comprehensive analysis of relationships of a node encompassing both immediate and higher-order neighbors, thereby enhancing event detection accuracy. Compared with existing techniques, the approach demonstrates superior performance in identifying distant events by efficiently capturing interdependencies among nodes.

## Figures and Tables

**Figure 1:**
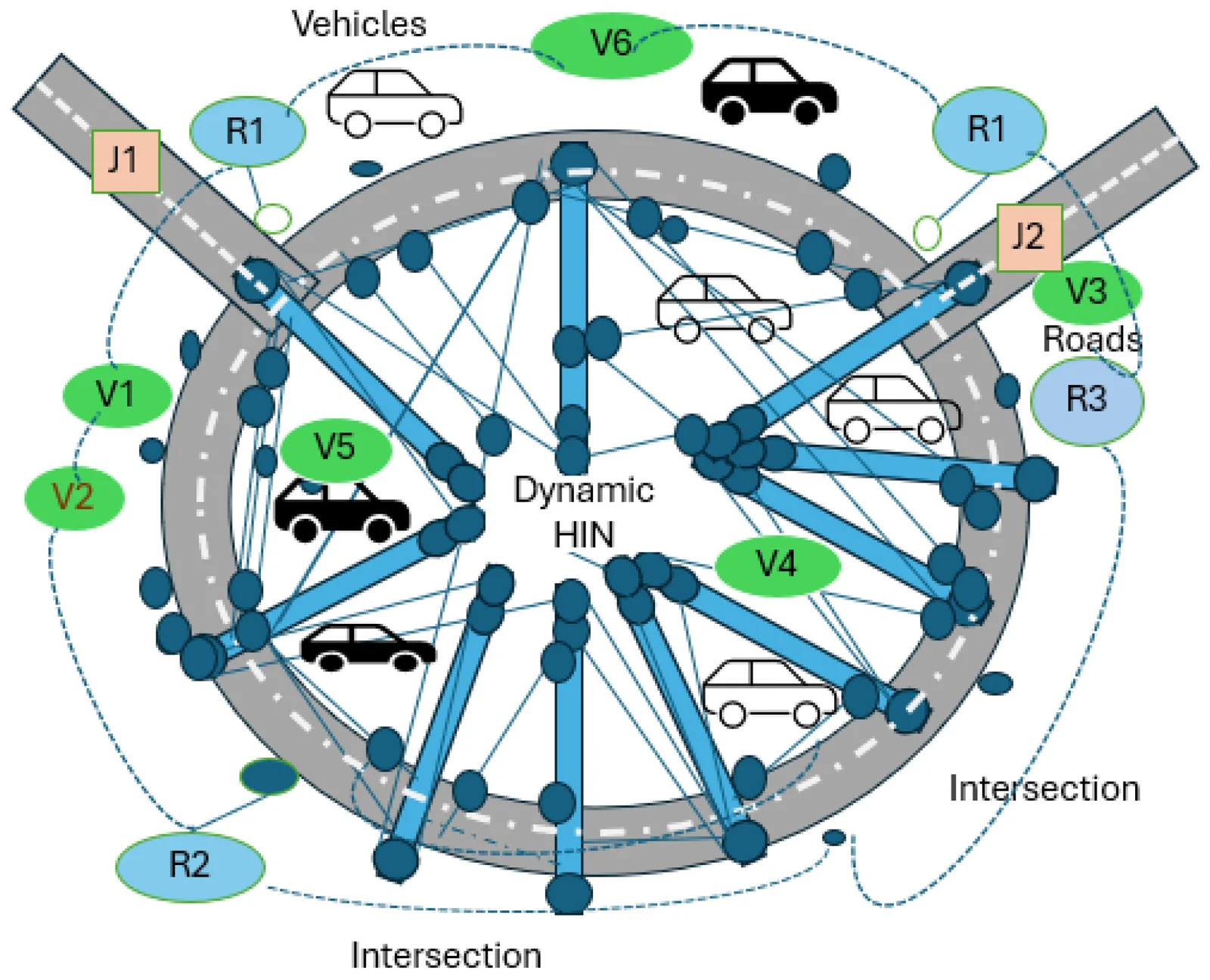
Two vehicles, V1 and V2 may navigate through the same junction, $J_{1}$ while $v_{1}$ and another, V3, might traverse a different junction, $J_{2}$. According to meta-path P1, V2 and V3 are neighbors of V1.

**Figure 2:**
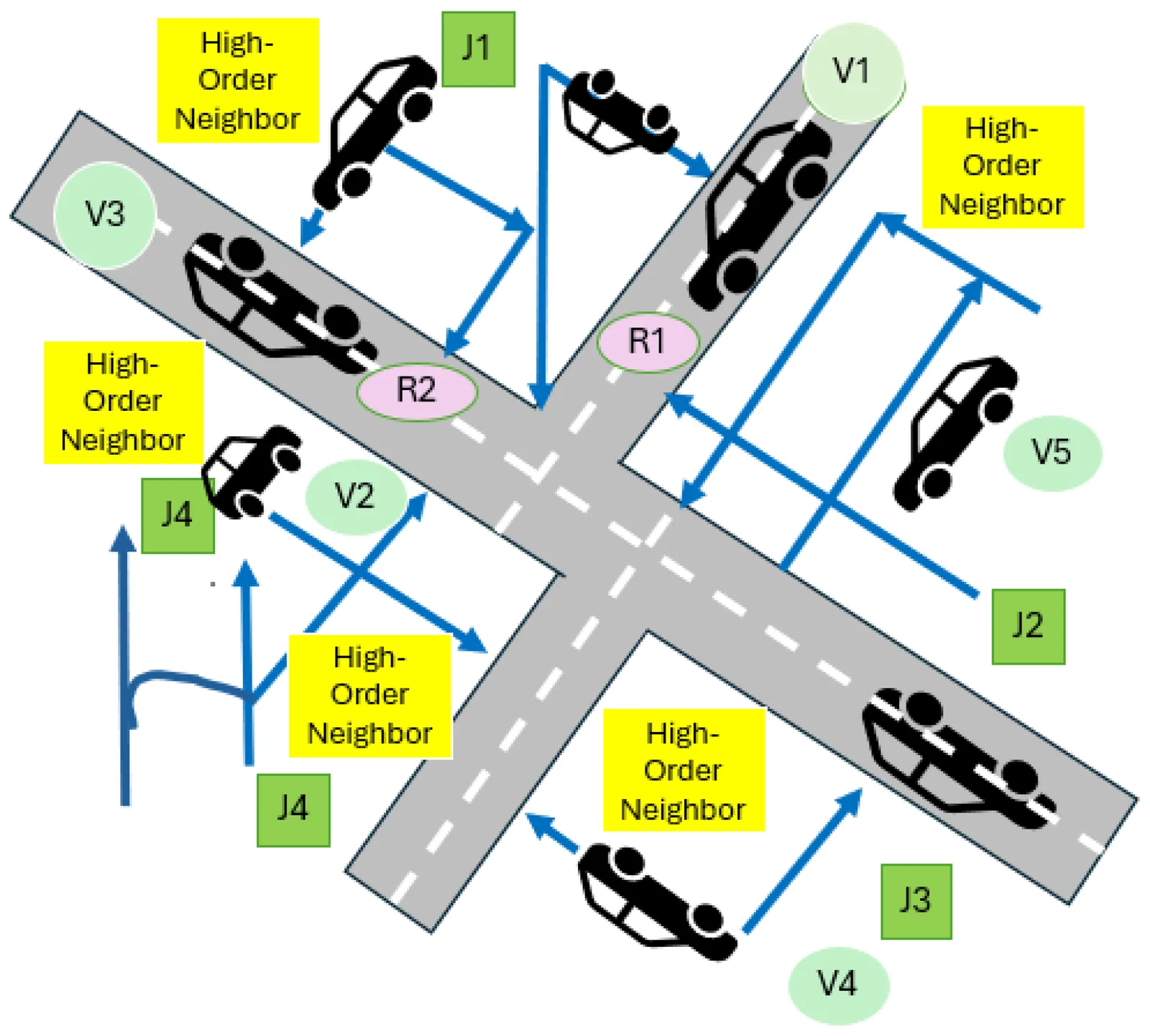
Meta-path for event detection on traffic.

**Figure 3:**
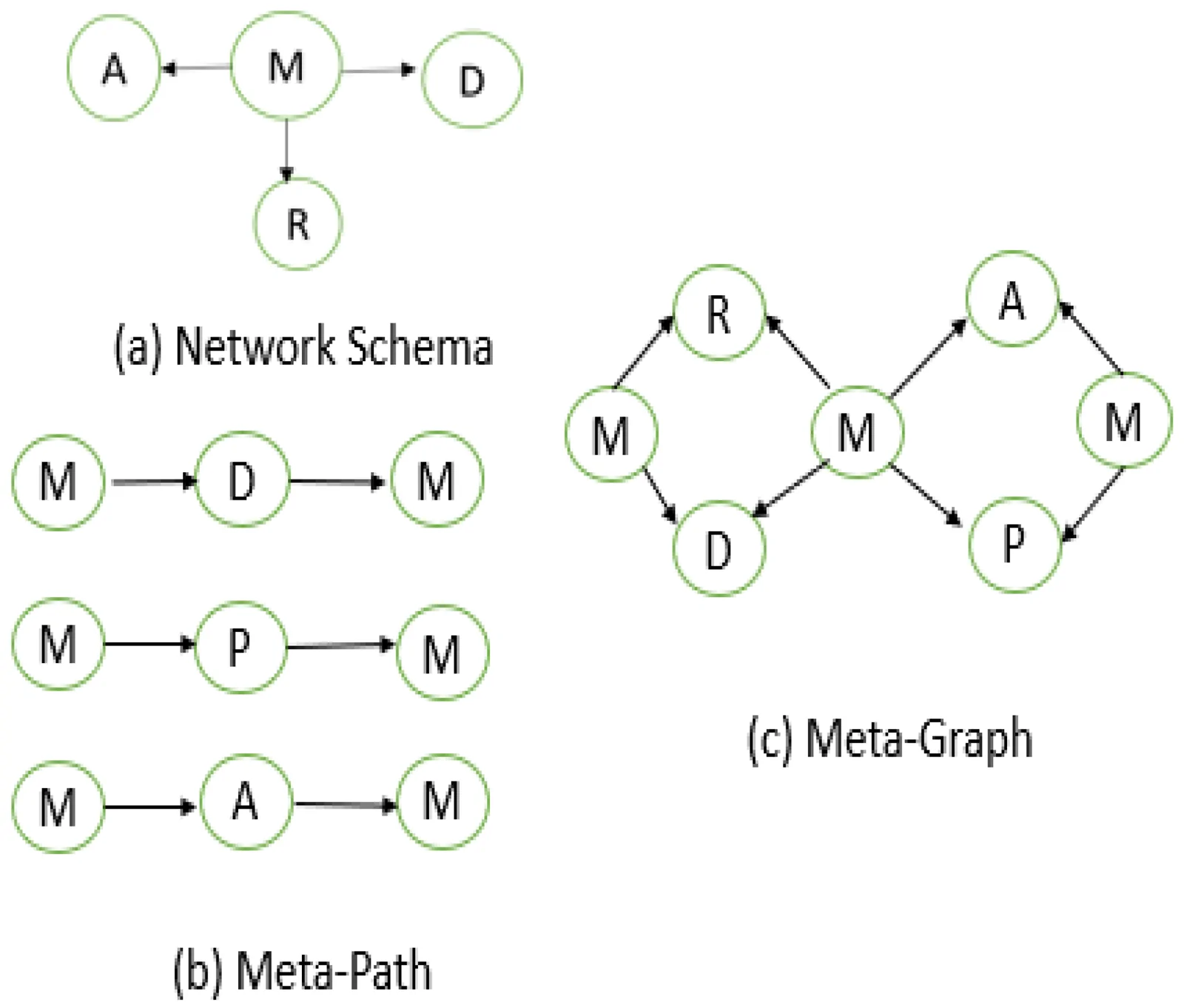
(**a**) Network schema, (**b**) meta-path, and (**c**) meta-graph. In this context, (A), (M), (D), and (R) represent Agent, Movement, Destination, and Route, respectively, for real-time traffic event detection.

**Figure 4:**
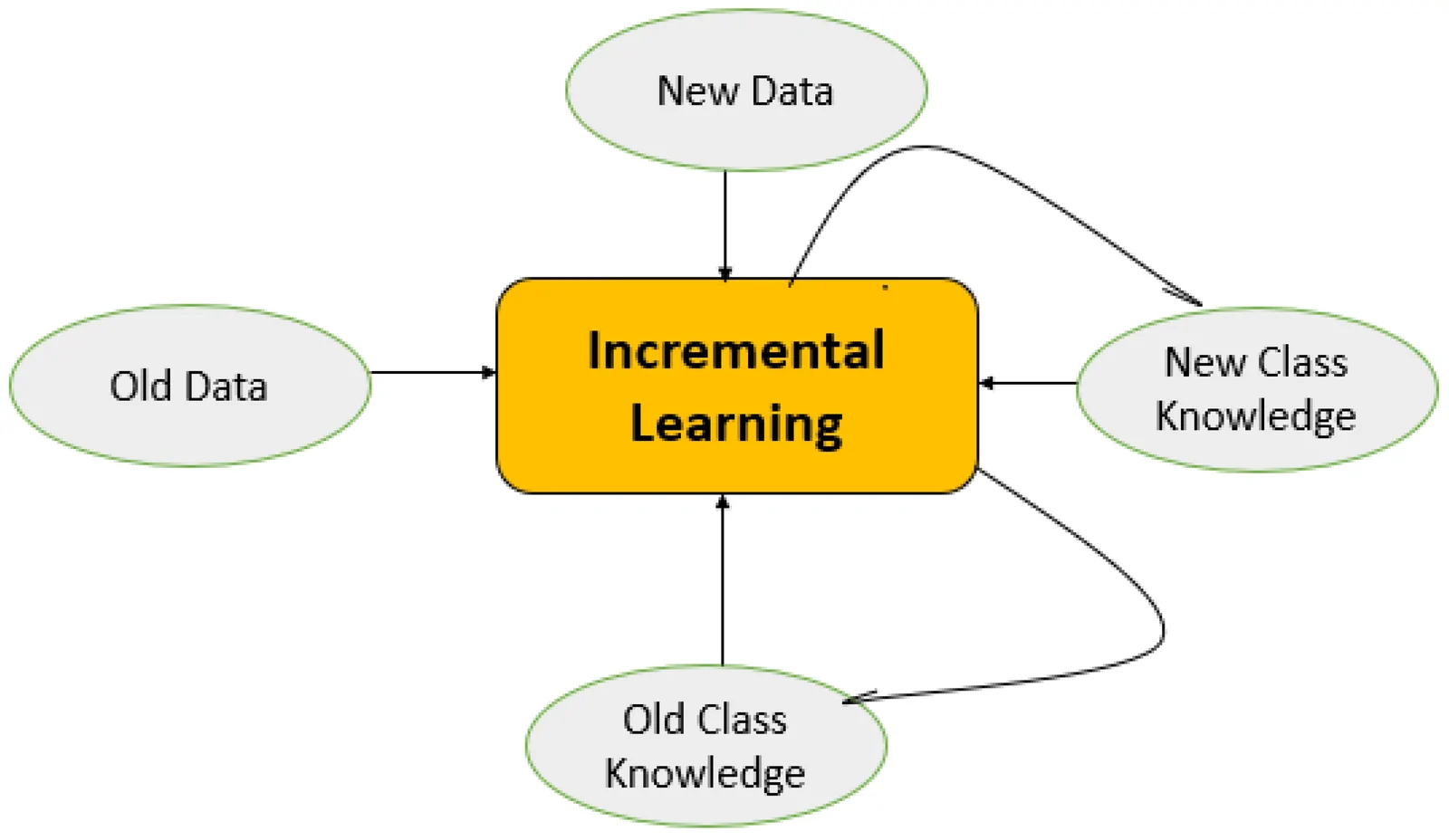
Incremental Learning Process.

**Figure 5:**
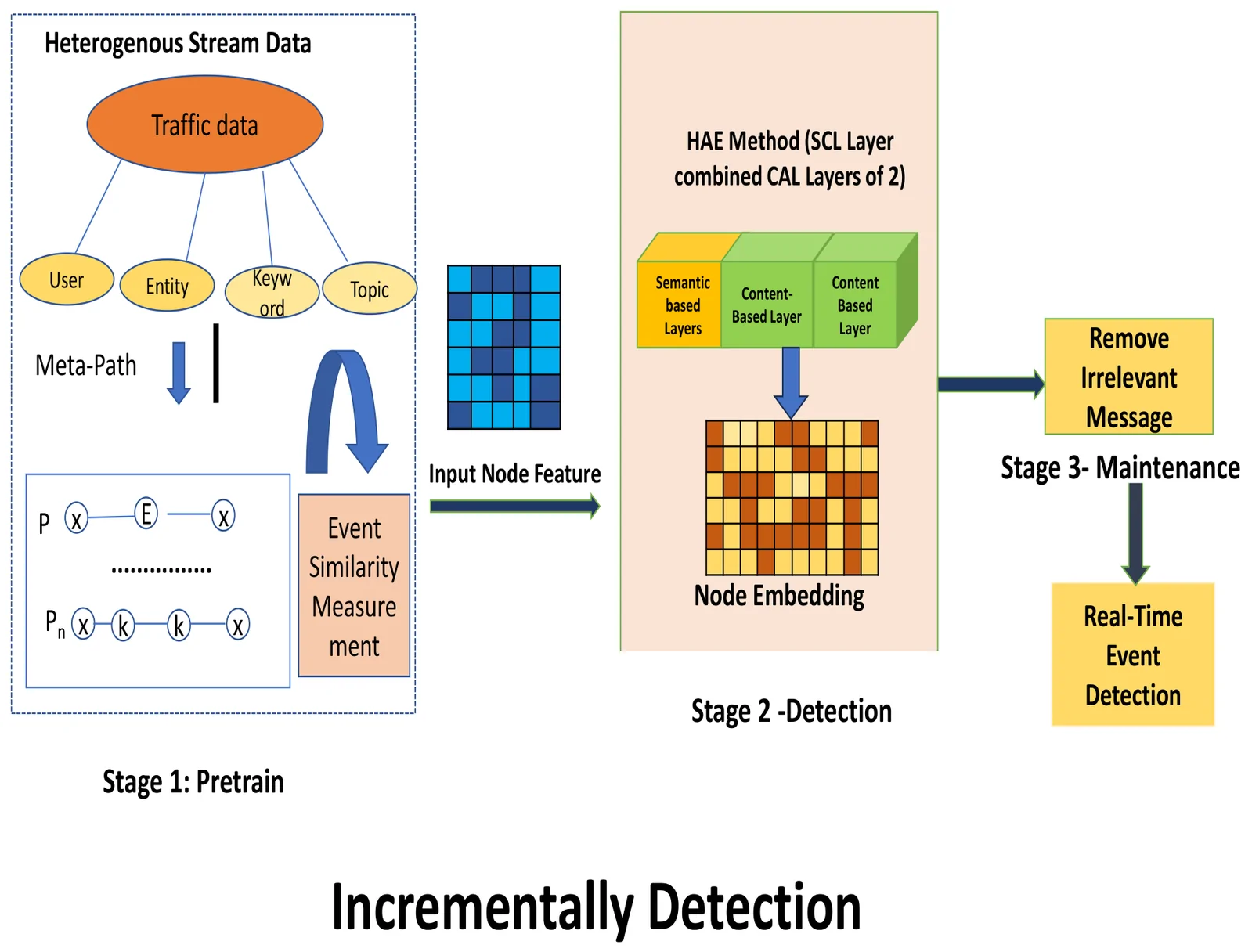
Block Diagram of Proposed Method.

**Figure 6:**
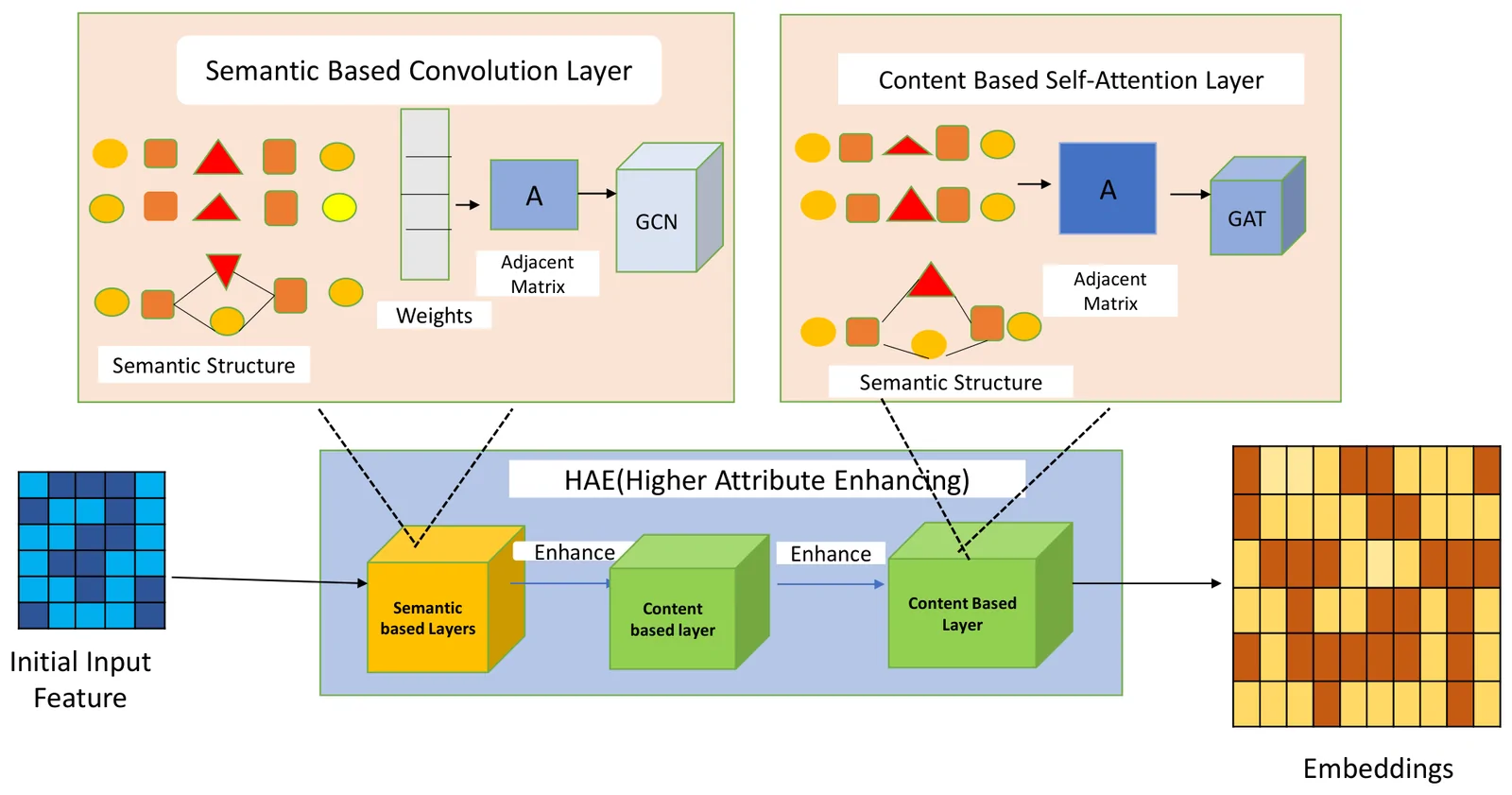
Working Model of Higher Attribute Enhancing (HAE).

**Figure 7:**
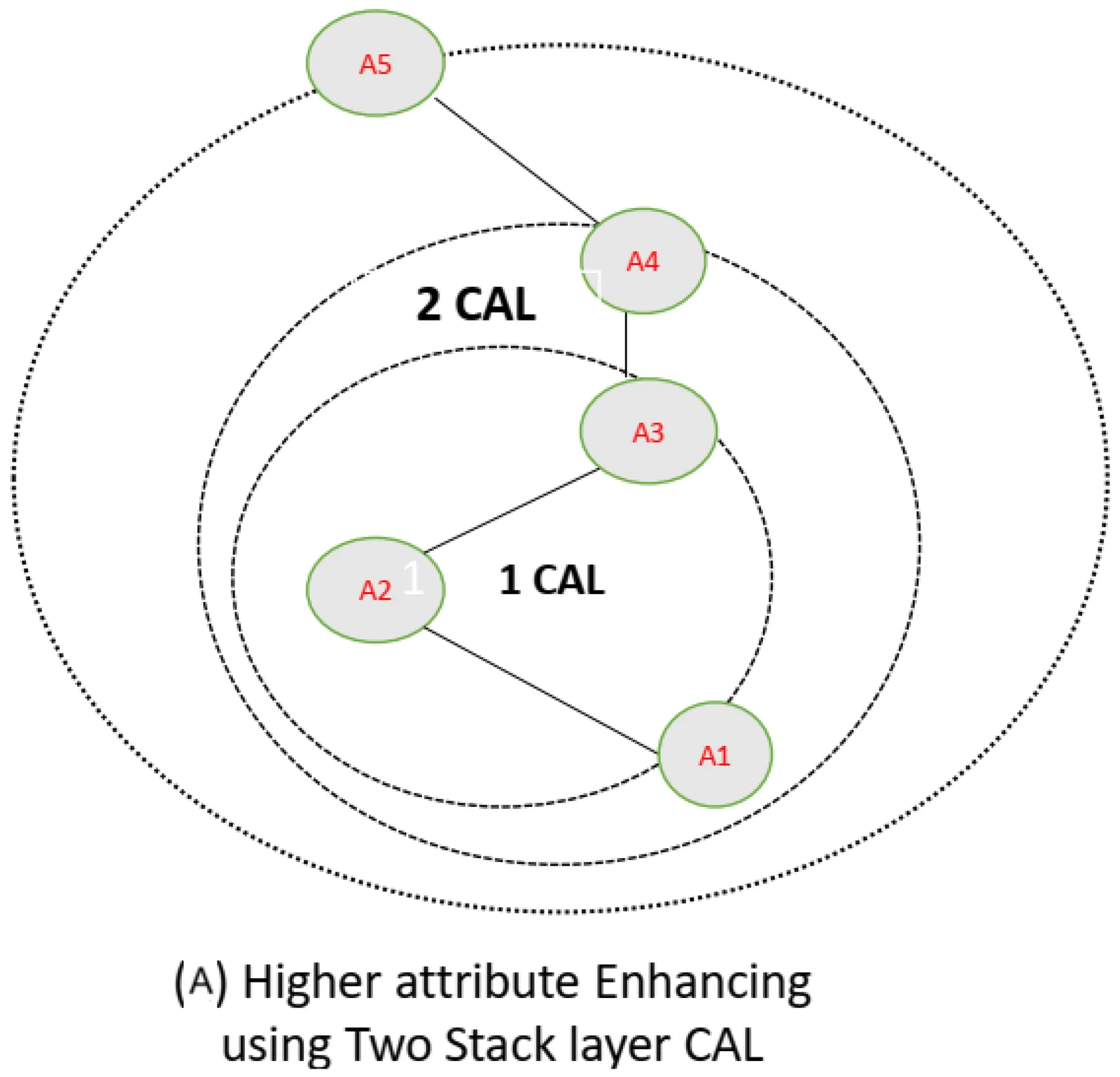
Higher attribute enhancement using a two-stack-layer CAL.

**Figure 8:**
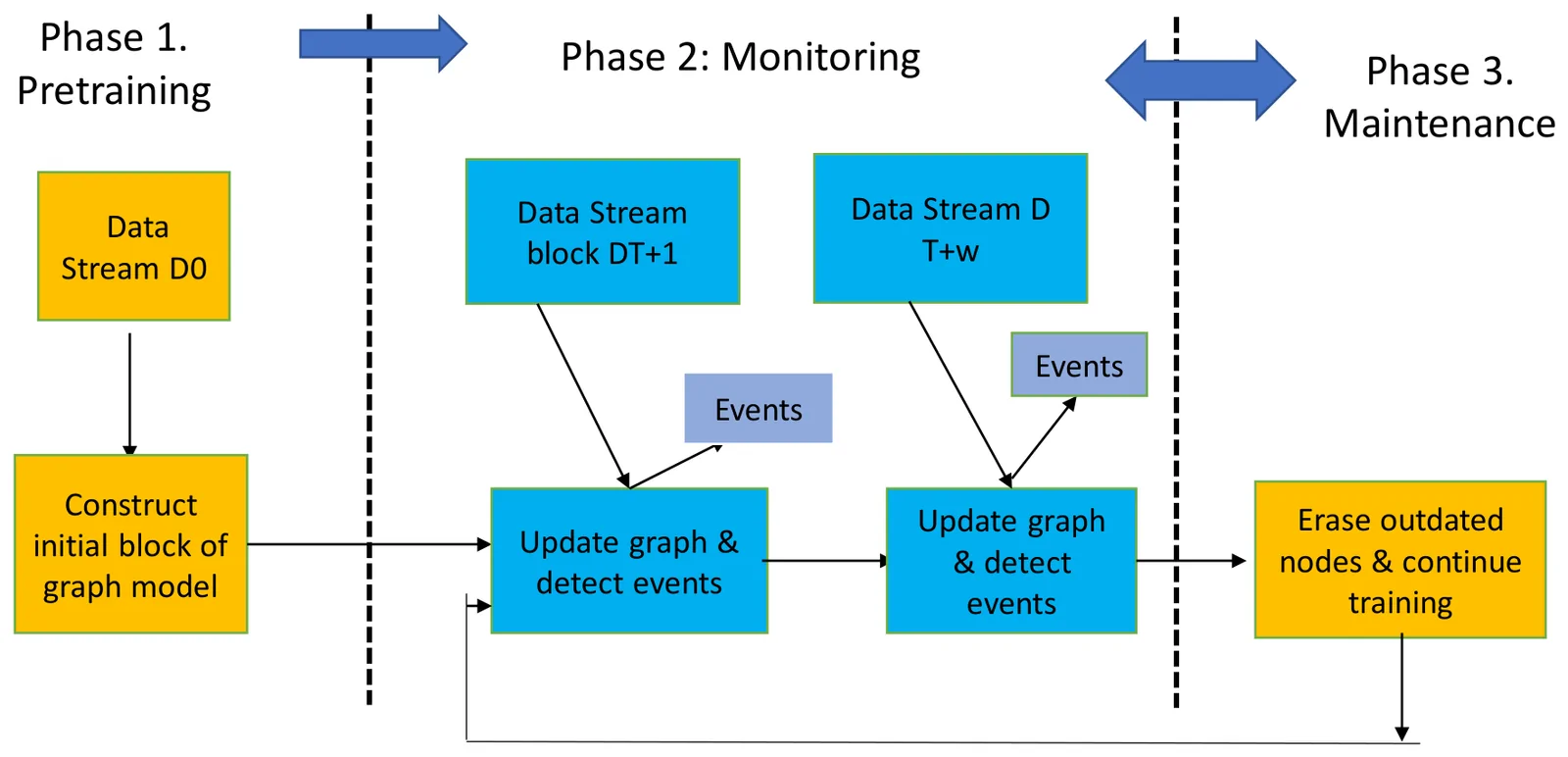
Incremental Learning Life Cycle.

**Figure 9:**
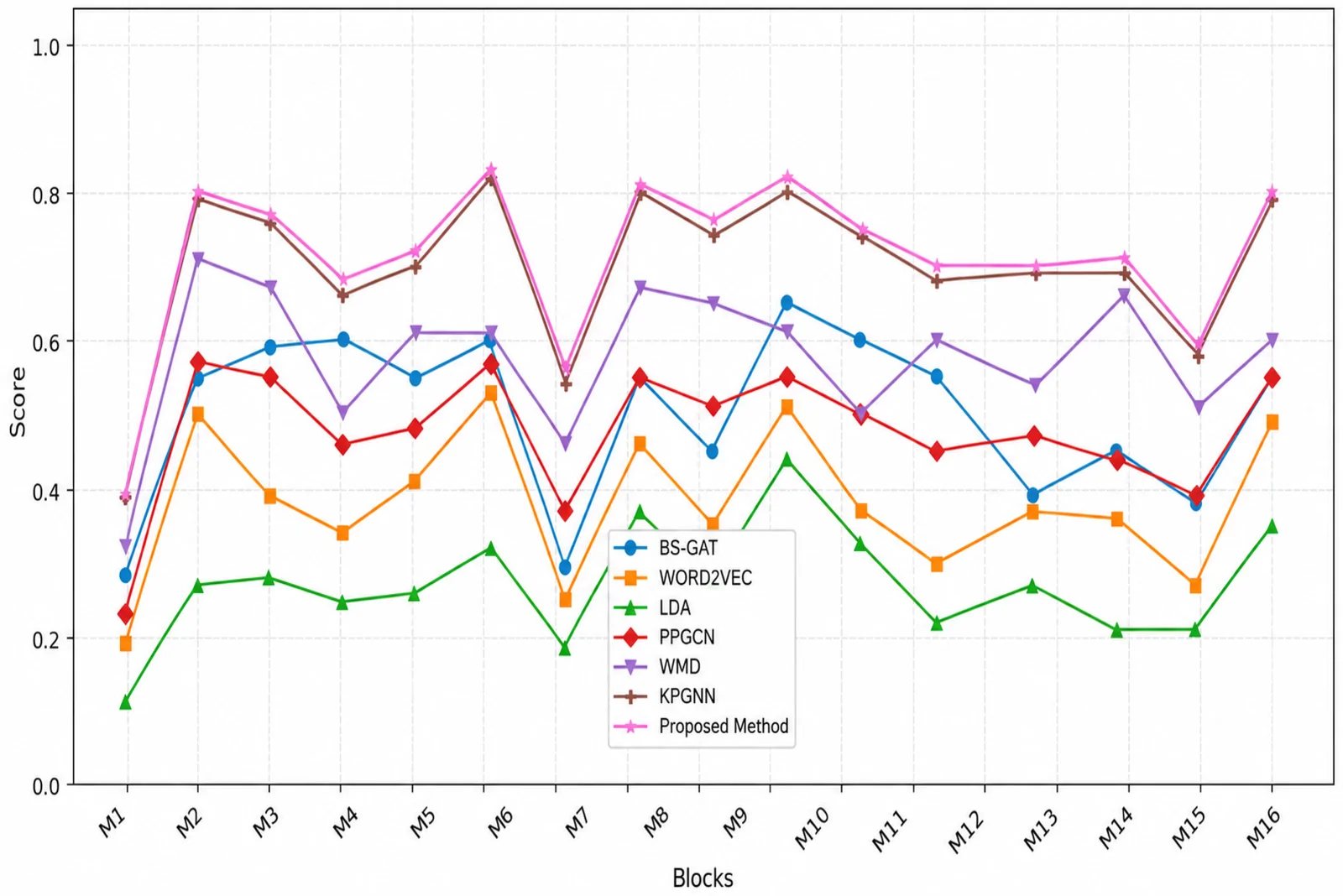
Comparison of Various Techniques Using Normalized Mutual Information (NMI).

**Figure 10:**
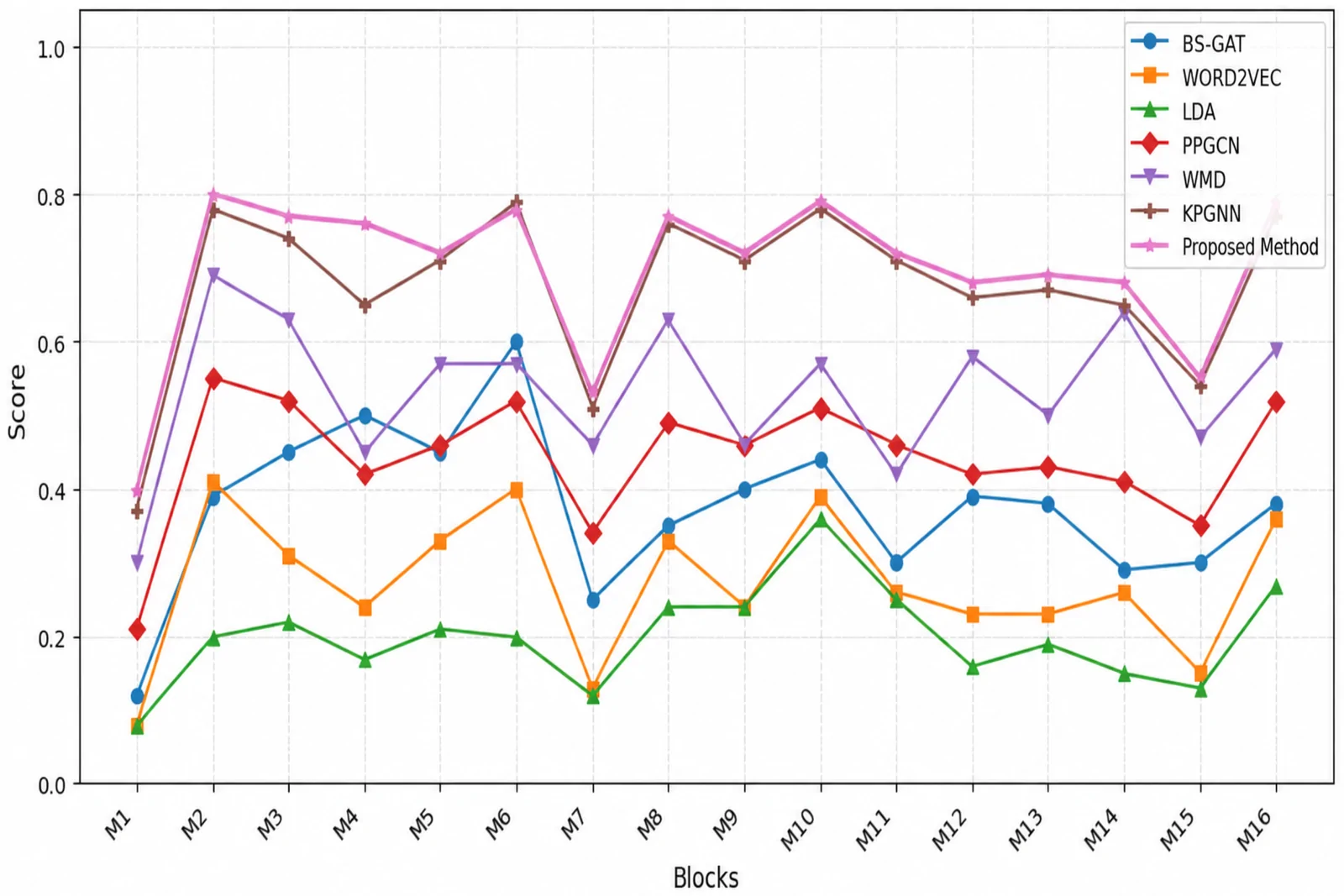
Comparison of Various Techniques Using Adjusted Mutual Information Values (AMI).

**Figure 11:**
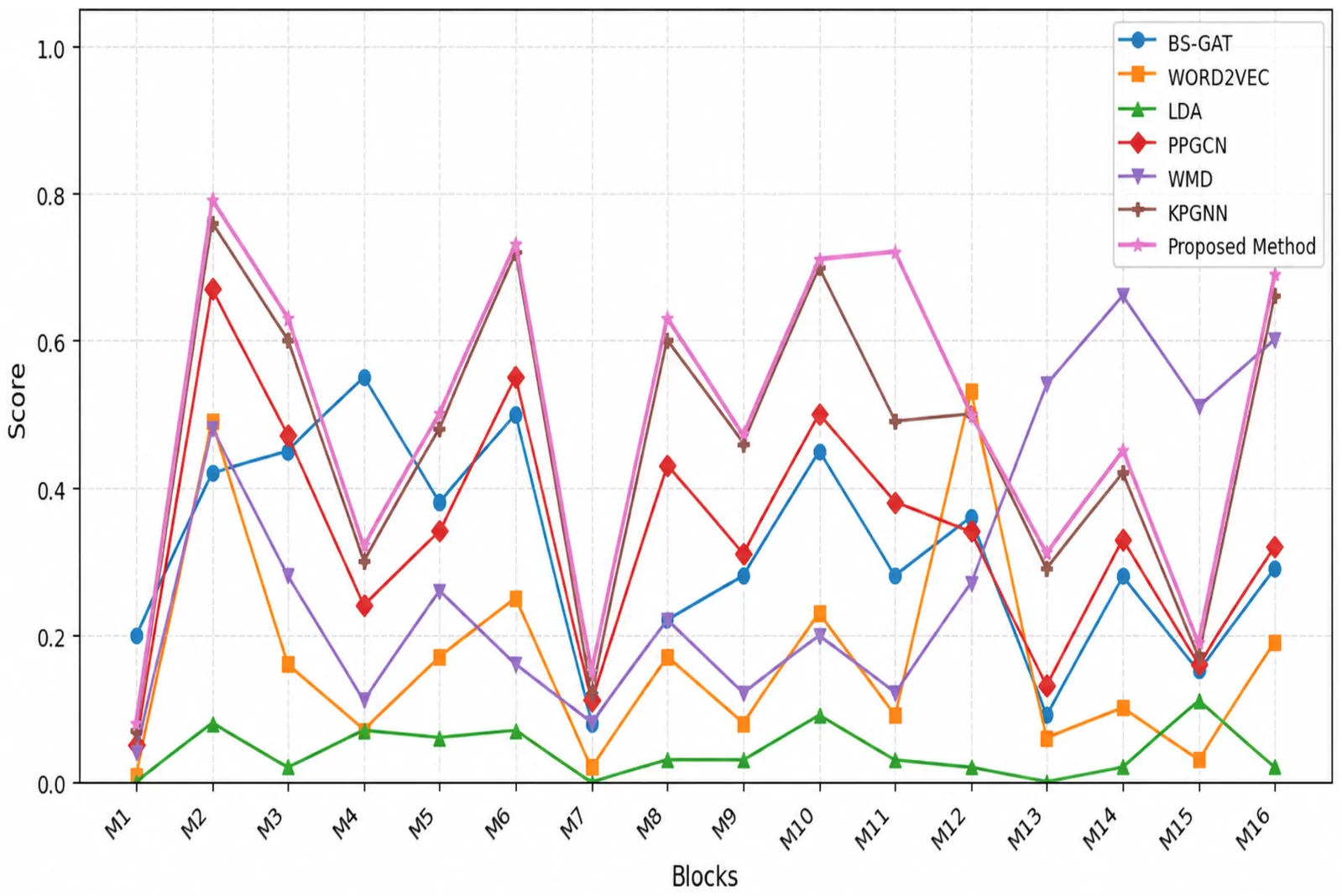
Comparison of Various Techniques Using Adjusted Rand Index Values (ARI).

**Figure 12:**
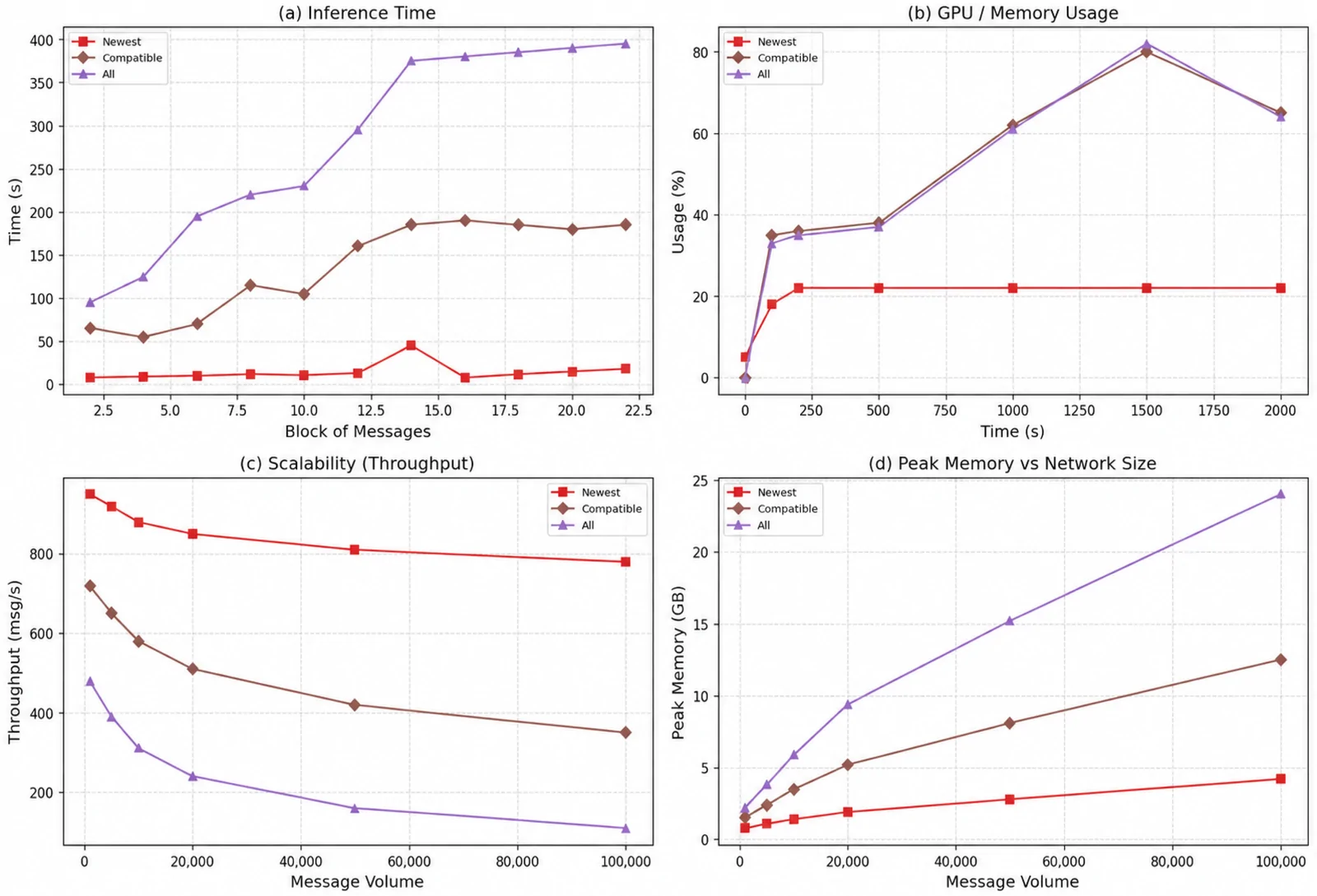
Ablation study.

**Table 1:** Symbols with Description.

Symbol	Description
$H^{i+1}$	Output node feature of the (*i* + 1)th layer
α	Activation function
*D*	Diagonal matrix
$y_{v}$	Control matrix at time *t*
*A*	Rich heterogeneous semantic information
$W^{I}$	Measurement noise
*H*	Independent attention mechanism
$g_{ij}$	Importance of $v_{tj}$’s representation to $v_{ti}$
$\alpha_{ij}$	Normalized attention coefficient
$N_{t}$	Set of first-order semantic neighbours of $v_{tj}$
$Wv_{ti}$	Weight matrix associated with node $v_{ti}$
$Wv_{tj}$	Weight matrix associated with node $v_{tj}$
$W^{h}$	Head-wise linear transformation matrix
$L_{T},\;L$	Loss functions
$V^{M}$	Measurement model
$A_{t}$	Total number of nodes

**Table 2:** Incremental Evaluation with Normalized Mutual Information (NMI) Across Blocks M1 to M16.

**Blocks**	**M1**	**M2**	**M3**	**M4**	**M5**	**M6**
BS-GAT [10]	0.28	0.55	0.59	0.60	0.55	0.60
WORD2VEC [22]	0.19	0.50	0.39	0.34	0.41	0.53
LDA [23]	0.11	0.27	0.28	0.25	0.26	0.32
PPGCN [25]	0.23	0.57	0.55	0.46	0.48	0.57
WMD [24]	0.32	0.71	0.67	0.50	0.61	0.61
KPGNN [21]	0.39	0.79	0.76	0.66	0.70	0.82
Proposed Method	0.39	0.80	0.77	0.68	0.72	0.83
**Blocks**	**M7**	**M8**	**M9**	**M10**	**M11**	**M12**
BS-GAT [10]	0.29	0.55	0.45	0.65	0.60	0.55
WORD2VEC [22]	0.25	0.46	0.35	0.51	0.37	0.30
LDA [23]	0.18	0.37	0.24	0.44	0.33	0.22
PPGCN [25]	0.37	0.55	0.51	0.55	0.50	0.45
WMD [24]	0.46	0.67	0.65	0.61	0.50	0.60
KPGNN [21]	0.54	0.80	.74	0.80	0.74	0.68
Proposed Method	0.56	0.81	0.76	0.82	0.75	0.70
**Blocks**	**M13**	**M14**	**M15**	**M16**		
BS-GAT [10]	0.39	0.45	0.38	0.55		
WORD2VEC [22]	0.37	0.36	0.27	0.49		
LDA [23]	0.27	0.21	0.21	0.35		
PPGCN [25]	0.47	0.44	0.39	0.55		
WMD [24]	0.54	0.66	0.51	0.60		
KPGNN [21]	0.69	0.69	0.58	0.79		
Proposed Method	0.70	0.71	0.59	0.80		

**Table 3:** Incremental Evaluation with Adjusted Mutual Information (AMI) Across Blocks M1 to M16.

**Blocks**	**M1**	**M2**	**M3**	**M4**	**M5**	**M6**
BS-GAT [10]	0.12	0.39	0.45	0.50	0.45	0.60
WORD2VEC [22]	0.08	0.41	0.31	0.24	0.33	0.40
LDA [23]	0.08	0.20	0.22	0.17	0.21	0.20
PPGCN [25]	0.21	0.55	0.52	0.42	0.46	0.52
WMD [24]	0.30	0.69	0.63	0.45	0.57	0.57
KPGNN [21]	0.37	0.78	0.74	0.65	0.71	0.79
Proposed Method	0.40	0.80	0.77	0.76	0.72	0.78
**Blocks**	**M7**	**M8**	**M9**	**M10**	**M11**	**M12**
BS-GAT [10]	0.25	0.35	0.40	0.44	0.30	0.39
WORD2VEC [22]	0.13	0.33	0.24	0.39	0.26	0.23
LDA [23]	0.12	0.24	0.24	0.36	0.25	0.16
PPGCN [25]	0.34	0.49	0.46	0.51	0.46	0.42
WMD [24]	0.46	0.63	0.46	0.57	0.42	0.58
KPGNN [21]	0.51	0.76	0.71	0.78	0.71	0.66
Proposed Method	0.53	0.77	0.72	0.79	0.72	0.68
**Blocks**	**M13**	**M14**	**M15**	**M16**		
BS-GAT [10]	0.38	0.29	0.30	0.38		
WORD2VEC [22]	0.23	0.26	0.15	0.36		
LDA [23]	0.19	0.15	0.13	0.27		
PPGCN [25]	0.43	0.41	0.35	0.52		
WMD [24]	0.50	0.64	0.47	0.59		
KPGNN [21]	0.67	0.65	0.54	0.77		
Proposed Method	0.69	0.68	0.55	0.79		

**Table 4:** Incremental Evaluation with Adjusted Rand Index (ARI) Across Blocks M1 to M16.

**Blocks**	**M1**	**M2**	**M3**	**M4**	**M5**	**M6**
BS-GAT [10]	0.20	0.42	0.45	0.55	0.38	0.50
WORD2VEC [22]	0.01	0.49	0.16	0.07	0.17	0.25
LDA [23]	0.00	0.08	0.02	0.07	0.06	0.07
PPGCN [25]	0.05	0.67	0.47	0.24	0.34	0.55
WMD [24]	0.04	0.48	0.28	0.11	0.26	0.16
KPGNN [21]	0.07	0.76	0.60	0.30	0.48	0.72
Proposed Method	0.08	0.79	0.63	0.32	0.50	0.73
**Blocks**	**M7**	**M8**	**M9**	**M10**	**M11**	**M12**
BS-GAT [10]	0.08	0.22	0.28	0.45	0.28	0.36
WORD2VEC [22]	0.02	0.17	0.08	0.23	0.09	0.53
LDA [23]	0.00	0.03	0.03	0.09	0.03	0.02
PPGCN [25]	0.11	0.43	0.31	0.50	0.38	0.34
WMD [24]	0.08	0.22	0.12	0.20	0.12	0.27
KPGNN [21]	0.12	0.60	0.46	0.70	0.49	0.50
Proposed Method	0.15	0.63	0.47	0.71	0.72	0.50
**Blocks**	**M13**	**M14**	**M15**	**M16**		
BS-GAT [10]	0.09	0.28	0.15	0.29		
WORD2VEC [22]	0.06	0.10	0.03	0.19		
LDA [23]	0.00	0.02	0.11	0.02		
PPGCN [25]	0.13	0.33	0.16	0.32		
WMD [24]	0.24	0.66	0.51	0.60		
KPGNN [21]	0.29	0.42	0.17	0.66		
Proposed Method	0.31	0.45	0.19	0.69		

**Table 5:** Comparative Analysis.

Blocks	Precision	Recall	F1
SHetGNN [26]	0.9734	0.9725	0.9791
SRGTNED [17]	0.793	0.799	0.796
CNN and LSTM [14]	0.976	0.962	0.972
Proposed Method	0.9843	0.9887	0.9855

## Data Availability

The datasets generated during and/or analysed during the current study are available from the corresponding author on reasonable request.
